# Foetal, neonatal and child vitamin D status and enamel hypomineralization

**DOI:** 10.1111/cdoe.12372

**Published:** 2018-03-01

**Authors:** Justin T. van der Tas, Marlies E.C. Elfrink, Annemieke C. Heijboer, Fernando Rivadeneira, Vincent W.V. Jaddoe, Henning Tiemeier, Josje D. Schoufour, Henriëtte A. Moll, Edwin M. Ongkosuwito, Eppo B. Wolvius, Trudy Voortman

**Affiliations:** ^1^ The Generation R Study Group Erasmus University Medical Center Rotterdam The Netherlands; ^2^ Department of Oral & Maxillofacial Surgery Special Dental Care and Orthodontics Erasmus University Medical Center Rotterdam The Netherlands; ^3^ Department of Clinical Chemistry Endocrine Laboratory VU University Medical Center Amsterdam The Netherlands; ^4^ Department of Epidemiology Erasmus University Medical Center Rotterdam The Netherlands; ^5^ Department of Internal Medicine Erasmus University Medical Center Rotterdam The Netherlands; ^6^ Department of Pediatrics Erasmus University Medical Center Rotterdam The Netherlands; ^7^ Department of Child and Adolescent Psychiatry/psychology Erasmus University Medical Center/Sophia Rotterdam The Netherlands; ^8^ Department of Psychiatry Erasmus University Medical Center Rotterdam The Netherlands

**Keywords:** 25‐hydroxyvitamin D, enamel biomineralization/formation, epidemiology, paediatric dentistry, permanent dentition, primary dentition, risk prediction

## Abstract

**Objectives:**

Recent literature suggested that higher vitamin D concentrations in childhood are associated with a lower prevalence of molar incisor hypomineralization (MIH). As tooth development already starts in utero, we aimed to study whether vitamin D status during foetal, postnatal and childhood periods is associated with the presence of hypomineralized second primary molars (HSPMs) and/or MIH at the age of six.

**Methods:**

Our study was embedded in the Generation R Study, a population‐based, prospective cohort from foetal life onwards in Rotterdam, the Netherlands. HSPMs and MIH were scored from intraoral photographs of the children at their age of six. Serum 25(OH)D concentrations were measured at three points in time, which resulted in three different samples; mid‐gestational in mothers’ blood (n = 4750), in umbilical cord blood (n = 3406) and in children's blood at the age of 6 years (n = 3983).

**Results:**

The children had a mean (±SD) age of 6.2 (±0.5) years at the moment of taking the intraoral photographs. After adjustment for confounders, no association was found between foetal 25(OH)D concentrations and the presence of HSPMs (OR 1.02 per 10 nmol/L higher 25(OH)D, 95% CI: 0.98‐1.07) or MIH (OR 1.05 per 10 nmol/L increase, 95% CI: 0.98‐1.12) in 6‐year‐olds. A higher 25(OH)D concentration in umbilical cord blood resulted in neither lower odds of having HSPM (OR 1.05, 95% CI: 0.98‐1.13) nor lower odds of having MIH (OR 0.95, 95% CI: 0.84‐1.07) by the age of six. Finally, we did not find higher 25(OH)D concentrations at the age of six to be associated with a significant change in the odds of having HSPM (OR 0.97, 95% CI: 0.92‐1.02) or MIH (OR 1.07, 95% CI: 0.98‐1.16).

**Conclusions:**

25(OH)D concentrations in prenatal, early postnatal and later postnatal life are not associated with the presence of HPSMs or with MIH at the age of six. Future observational research is required to replicate our findings. Furthermore, it is encouraged to focus on identifying other modifiable risk factors, because prevention of hypomineralization is possible only if the causes are known.

## 1 INTRODUCTION

Dental enamel hypomineralization is an anomaly of dental enamel in which the affected enamel contains less mineral than sound enamel and is more susceptible to caries.^1^, ^2^, ^3^ This anomaly can be divided into hypomineralization of second primary molars, called hypomineralized second primary molars (HSPMs), and hypomineralization of permanent first molars, called molar incisor hypomineralization (MIH).^3^, ^4^, ^5^ In patients with MIH incisors of the upper jaw can also be involved and in rare cases incisors of the lower jaw.^3^ Although hypomineralization is not restricted to those few index teeth and can be diagnosed in any tooth of both dentitions, a patient can only be diagnosed with HSPM/MIH if he or she has at least one affected second primary molar or first permanent molar, respectively.^6^ The prevalence of HSPMs is about 4.9% in 6‐year‐old Dutch children.^7^ For MIH, the prevalence ranges between 8% and 19% among Dutch and Scandinavian children aged six to thirteen years.^3^, ^5^, ^7^, ^8^ Children with HSPM have a higher chance of developing MIH.^9^, ^10^ Identifying modifiable risk factors is important to prevent development of dental enamel hypomineralization in children.

Several early life risk factors for HSPM and MIH have been identified. For HSPM, maternal alcohol consumption during pregnancy, low birth weight and fever during the first year of life are mentioned.^11^ Other illnesses in early life and the use of antibiotics were proposed as risk factors for MIH.^12^, ^13^ The exact aetiology of dental enamel hypomineralization, however, remains unclear.^4^, ^11^, ^12^, ^13^, ^14^ In the search to unravel the aetiology of dental hypomineralization, a recent study of Kühnisch et al^15^ showed that higher serum 25‐hydroxyvitamin D (25(OH)D) concentrations were correlated with less MIH and dental caries in 1048 German children at age ten. To our knowledge, this is the only study to have examined 25(OH)D and dental enamel hypomineralization. Several other studies examined vitamin D in relation to caries and generally observed that vitamin D supplementation in early life may be preventative for dental caries, as reviewed by Hujoel et al^16^

The main function of vitamin D is to maintain plasma calcium concentrations at a constant level, which is important for healthy bone development and increasing evidence suggests also for healthy tooth development.^17^, ^18^ Vitamin D stimulates mineralization of dental enamel and bone by binding to receptors that are expressed in both dental cells and bone cells.^19^, ^20^ Because vitamin D is important in the mineralization of these tissues, it is noteworthy that we recently discovered that lower bone mass is associated with the presence of HSPM but not with MIH in 6‐year‐old children.^21^ Our hypothesis is that this association could be explained by differences in 25(OH)D status between children, affecting mineralization of dental enamel and bone.

A limitation of the previous study of Kühnisch et al^15^ was that information on vitamin D status of the children was only available at 10 years of age, whereas tooth development and enamel mineralization already start earlier in life.^12^, ^22^ Accordingly, we aimed to replicate and extend these previous analyses by examining whether 25(OH)D concentrations during foetal life, early postnatal life and childhood are associated with HSPMs and/or MIH in 6‐year‐old children. Based on the previous literature, our hypothesis was that children, affected by HSPM or MIH, have significant lower 25(OH)D concentrations during earlier phases in their life than children with unaffected teeth.

## 2 METHODS

### 2.1 Study design and population

**Figure 1:**
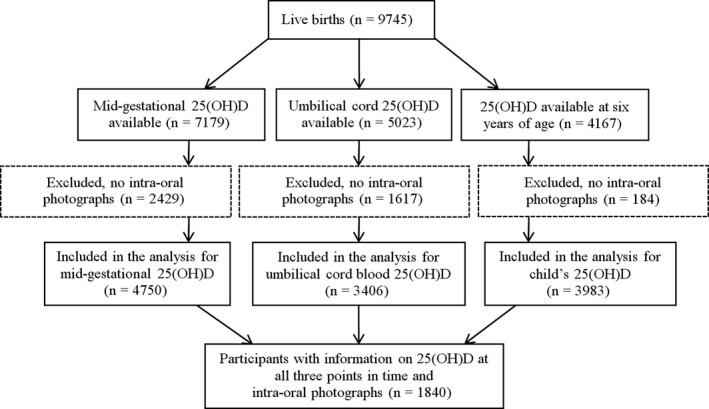
Flow chart of participants

The analysis was embedded in the Generation R Study, a population‐based, prospective cohort from foetal life onwards in Rotterdam, the Netherlands.^23^ Pregnant women living in the study area with a due date between April 2002 and January 2006 were eligible for enrolment. We enrolled 9778 mothers, who gave birth to a total of 9745 live‐born children. The study has been approved by the Medical Ethics Committee of Erasmus Medical Center, Rotterdam (MEC 198.782/2001/31). Written informed consent was obtained from parents of all participants. Concentrations of 25(OH)D were measured at three points in time; at mid‐gestation (18‐25 weeks of pregnancy) in 7179 mothers, after birth from a blood sample of the umbilical cord in 5023 children and at 6 years of age (mean 6.2 [range 4.9‐9.1]) in 4167 children. Intraoral photographs were made of 6325 children during the same visit at the age of six. The number of children with data on both 25(OH)D and intraoral photographs ranged from 3406 to 4750 for the different analyses (Figure 1).

### 2.2 HSPM and MIH diagnoses

To visualize HSPM and MIH, an intraoral camera was used (Poscam USB intraoral [Digital Leader PointNix] or Sopro 717 [Acteon] autofocus camera, 640 × 480 pixels). The minimal scene illumination of both cameras was 3.0 lx (F1.4). During the data collection period, pictures of the teeth were taken by six trained nurses, twelve dental students and six PhD students. A paediatric dentist (ME) gave them a presentation about the how and why of taking the dental photographs and repeated that each half a year. Before the employees/students were allowed to make photographs themselves, they had to accompany an experienced employee/student for a day and learned how to make high‐quality photographs. Afterwards, a paediatric dentist (ME) evaluated all photographs within 2 or 4 weeks. If she found the quality to be too low, she further instructed the respective employee/student on how to improve their quality or she trained them individually. Before taking the photographs, the children had to brush their teeth and excess saliva was removed with a cotton roll. Photographs were scored by a paediatric dentist (ME) on the presence of HSPM and MIH using the European Academy of Pediatric Dentistry (EAPD)criteria.^24^ After completion of the data collection period, the same paediatric dentist (ME) re‐evaluated the photographs of 649 children (10%) with a minimal time gap of 6 weeks. This resulted in a kappa for the intraobserver agreement of 0.82 for HSPM and 0.85 for MIH.^21^ A second paediatric dentist (JV) re‐evaluated the photographs of 648 children (10%). The kappa's for the interobserver agreement for ME and JV were 0.60 for HSPM and 0.69 for MIH.^10^, ^21^ JV evaluated the photographs only once. Hence, we were not able to calculate a kappa value for the intraobserver agreement of this examiner. ME and JV had a calibration session each 2 or 3 months. Before this session, ME randomly chose a couple photographs and discussed them together with JV. As photographs were taken at the age of six, not all children had their permanent first molars yet, resulting in a smaller number of children with data on MIH than HSPM. Children without data on MIH were on average younger (mean age 6.00 vs 6.41 years), were more often male (52.7% vs 44.7%) and more often had a Dutch or other Western background (68.3% vs 59.8%) than children with data on MIH (Table S1).

### 2.3 25(OH)D measurement

Maternal venous blood samples were collected during mid‐pregnancy at a median gestational age of 20.4 weeks (95% range 18.5‐23.4). After delivery, midwives or obstetricians collected cord blood from the umbilical vein at a median gestational age of 40.1 weeks (95% range 36.7‐42.3). Blood samples of the children were collected at the research centre at the 6‐year visit. Concentrations of 25(OH)D in these samples were analysed in two different laboratories.

25(OH)D concentrations in maternal blood samples and in umbilical cord blood were measured at the Eyles Laboratory of the Queensland Brain Institute, University of Queensland, Australia. Samples were quantified using isotope dilution liquid chromatography/tandem mass spectrometry (LC‐MS/MS). The method limit of quantification was 6 nmol/L and interassay imprecision was <11%.^25^

Vitamin D status of children's blood samples was measured at the Endocrine Laboratory of the VU University Medical Center, Amsterdam, the Netherlands as described in detail previously.^26^ Briefly, 25(OH)D was measured using isotope dilution online solid phase extraction LC‐MS/MS, a similar method as used for the foetal sample. The limit of quantitation was 4.0 nmol/L; intra‐assay coefficient of variation was <6%, and interassay coefficient of variation was <8% for concentrations between 25 and 180 nmol/L.^26^ This method was perfectly aligned with the reference methods.^27^

A cross‐validation in 31 umbilical cord and pregnancy blood samples that were analysed in both laboratories showed an excellent correlation between both methods (*r* = .99). The Passing & Bablok regression analysis resulted in 25(OH)D_Eyles_ = 0.93* 25(OH)D_VUmc_ + 0.3 nmol/L. This means that a small calibration difference of about 7% exists between the two LC‐MS/MS methods. As both assays show some interassay variation, we decided not to correct for this small difference.

We categorized 25(OH)D concentrations: ≥75 nmol/L (optimal), 50 to <75 nmol/L (sufficient), 25 to <50 nmol/L (deficient) and <25 nmol/L (severely deficient) on the basis of recommendations and cut‐offs used in previous studies.^26^, ^28^

Measuring the 25(OH)D concentrations at three different time points and assessing hypomineralization at one time point resulted in four different subsets of the population; three subsets with a 25(OH)D measurement at one point in time and dental data; and one subset with measurements at all three points in time and dental data. These subsets were highly comparable in terms of population characteristics (Table S2).

### 2.4 Covariates

Maternal age, educational level (low, mid‐low, mid‐high or high), parity, folic acid supplement use before/during pregnancy (start 1st 10 weeks, start periconceptional or never) and household income (<2000, 2000‐3300, >3300 Euros/month) were assessed at enrolment in the study (ie, during pregnancy) using questionnaires. Maternal smoking and alcohol consumption during pregnancy were assessed in each trimester of pregnancy and categorized into never, until pregnancy was known, or continued. Information on child's birth weight was acquired from medical records and hospital registries. Low birth weight was defined as a birth weight below 2500 grams. Children's ethnicity was defined based on birth country of both parents^29^ and categorized into Western (Dutch, other European, American and Oceanian), Moroccan and Turkish, African (Surinamese‐Creole, Antillean, Cape Verdean and other African), or Asian (Indonesian, other Asian and Surinamese‐Hindustani) on the basis of expected similarities in skin colour.^26^ Frequency of fever in the first year of life was assessed at age 12 months with questionnaires. During the research centre visit at the child's age of 6 years, we measured length and weight of the child. At the 6‐year follow‐up, we assessed duration of television watching (<2/≥2 h/d) and playing outside during daytime (<2/≥2 h/d) with questionnaires. At the 6‐year follow‐up, we re‐assessed household income (<2000, 2000‐3200 or >3200 Euros/month) and maternal educational level.^30^ For all blood sample analyses, we kept a record of the month and season of the year in which blood was drawn.

### 2.5 Statistical analyses

First, we constructed three binary logistic regression models in which having HSPMs at the age of six (yes/no) was defined as the outcome (dependent variable) and the foetal serum 25(OH)D concentrations were included as a predictor (independent variable). Foetal serum 25(OH)D concentrations were included as both a categorical variable and as a continuous variable per 10 nmol/L. The categories were compared to an optimal serum concentration of ≥75 nmol/L (reference category). Model 1 adjusted only for the child's sex, gestational age at blood withdrawal, mother's age and BMI before pregnancy. Model 2 additionally adjusted for variables that were associated with HSPMs in the Generation R Study population.^11^ In model 3, we added variables that were associated with serum 25(OH)D concentrations in our study population.^26^ We followed the same approach for studying the association between MIH (outcome) and foetal 25(OH)D serum concentrations (predictor). Moreover, we made use of the same models to study the association between HSPM and MIH as outcomes and cord blood serum 25(OH)D concentrations as predictor. For the approach in which the child's serum 25(OH)D concentrations at the age of six was used as a predictor, minor modifications in the model were made as follows: Model 1 was adjusted for child's sex, age, weight and length, model 2 did not change, and model 3 was adjusted for household income and maternal educational level at the child's age of six instead of at enrolment, and child's watching television and playing outside were added because these factors have been shown to be important for children's vitamin D status.^27^

To be able to compare results of foetal, birth and childhood 25(OH)D, we repeated the analyses in a subgroup with data available on 25(OH)D at all three time points (n = 1840, Figure 1). We tested for statistical interaction between vitamin D status and children's age, sex and ethnicity separately in model 3. Multicollinearity was evaluated but was found not to be a problem in our models, because the tolerance statistic exceeded 0.20 for all variables. Moreover, we examined whether we could assume 25(OH)D levels to be linear to the logit using natural cubic splines (degrees of freedom = 3). Missing data of covariates were handled by applying multiple imputation (n = 10 imputations).^31^ The pooled odds ratios (ORs) and 95% confidence intervals (95% CIs) were derived from pooling the results of the ten imputed datasets. Effect estimates were similar to the results of analyses of the original data, therefore, we only report pooled results after the imputation procedure. SPSS version 22.0 for Mac (IBM Corp, Armonk, NY, USA) was used for all analyses and a two‐sided *P*‐value of <.05 was considered to be statistically significant.

The STROBE Guidelines were used to ensure adequate reporting of this observational study.^32^

## 3 RESULTS

Children in our sample had a mean (±SD) age of 6.2 (±0.5) years at assessment (Table 1). Half of all participants had optimal or sufficient 25(OH)D serum concentrations above 50 nmol/L (50.1%) at the mid‐gestational period; 26.5% were deficient, and 23.4% were severely deficient in 25(OH)D. About 10% of the children had incomplete image sets or too low‐quality photographs to score HSPMs. To score MIH, 62.5% of the children had incomplete image sets (ie, no erupted first permanent molars) or too low‐quality photographs. The prevalence of HSPM in this population was 8.9% (381 out of 4278), and it was 8.1% (146 out of 1780) for MIH.

**Table 1:** Maternal and child characteristics in the total group of children with foetal 25(OH)D concentration measurements^a^, ^b^

Maternal characteristics	Total group (n = 4750)	Child characteristics	Total group (n = 4750)
Age (y)	30.4 ± 5.0	Age (y)	6.2 ± 0.5
Length (cm)	168 ± 7.4	Male (%)	49.7 (2359)
BMI (kg/cm^2^)	24.7 ± 4.4	Birth weight (kg)	3.4 ± 0.6
Parity (% (n))		Low birth weight (% (n))	5.0 (236)
Nulliparous	57.4 (2727)	Weight (kg)	23.2 ± 4.2
Primi‐ or multiparous	42.6 (1991)	Length (cm)	119 ± 5.9
Missing	0.7 (32)	Fever in first year of life (% (n))	
Educational level (% (n))		Yes	82.0 (2573)
High	28.9 (1165)	No	18.0 (566)
Mid‐high	27.6 (1115)	Missing	33.9 (1611)
Mid‐low	31.4 (1268)	Ethnicity (% (n))	
Low	12.1 (488)	Dutch and other Western	65.0 (3035)
Missing	15.0 (714)	Moroccan and Turkish	14.0 (653)
Household Income/month (% (n))		African	14.8 (691)
>3200 euro	49.8 (1912)	Asian	6.2 (288)
2000‐3200 euro	26.1 (1000)	Missing	1.8 (83)
<2000 euro	24.1 (925)	Watching television (% (n))^c^	
Missing	19.2 (913)	<2 h/d	80.5 (2621)
Alcohol use during pregnancy (% (n))		≥2 h/d	19.5 (636)
Never	45.1 (1887)	Missing	21.8 (910)
Alcohol use until pregnancy was known	14.3 (598)	Playing outside during daytime (% (n))^c^	
Continued	40.6 (1697)	≥2 h/d	23.0 (704)
Missing	12.0 (568)	<2 h/d	77.0 (2358)
Folic acid use during pregnancy (% (n))		Missing	26.5 (1105)
Start 1st 10 weeks	32.2 (1170)	Season of blood withdrawal (% (n))	
Start periconceptional	43.5 (1580)	Winter	24.0 (1140)
Never	24.2 (879)	Spring	28.3 (1343)
Missing	23.6 (1121)	Summer	22.3 (1060)
		Fall	25.4 (1207)
		25(OH)D Concentration (% (n))	
		Optimal + Sufficient (≥50 nmol/L)	50.1 (2381)
		Deficient (25‐50 nmol/L)	26.5 (1258)
		Severely deficient (<25 nmol/L)	23.4 (1111)
		Evaluable photographs (% (n))	
		HSPM	90.0 (4278)
		MIH	37.5 (1780)
		Prevalence HSPM (% (n))^d^	8.9 (381)
		Prevalence MIH (% (n))^e^	8.2 (146)

a Values are means ± SDs for continuous variables and percentages for categorical variables based on the number of valid cases.

b For the categorical variables, the percentage of missing data is shown.

c Based on group of children with childhood 25(OH)D concentration measurements (n = 4167).

d Based on group of children with evaluable photographs for HSPM (n = 4278).

e Based on group of children with evaluable photographs for MIH (n = 1780).

The results of the logistic regression analyses with mid‐gestational serum 25(OH)D concentration as a predictor and dental enamel hypomineralization as the outcome are shown in Table 2. In model 1, children from mothers with severely deficient mid‐gestational 25(OH)D concentrations had significantly lower odds of having HSPMs (OR, 0.67; 95% CI, 0.50‐0.91) than those from mothers with sufficient or optimal 25(OH)D concentrations. Similar associations were observed for 25(OH)D as a continuous variable. However, no association with HSPMs remained statistically significant in models 2 and 3. The foetal 25(OH)D concentration was not associated with the presence of MIH in children.

**Table 2:** Associations of mid‐gestational serum 25(OH)D concentrations with HSPM and MIH

	Mid‐gestational Serum 25(OH)D concentrations			
	≥50 nmol/L (Sufficient to Optimal)	25‐50 nmol/L (Deficient)	<25 nmol/L (Severely Def.)	Per 10 nmol/L
HSPM (n* *=* *4278) (Yes vs No)	n* *=* *2184 (222 vs 2184)	n* *=* *1126 (96 vs 1030)	n* *=* *968 (63 vs 905)	n* *=* *4728 (381 vs 3897)
OR (95% CI)				
Model 1^a^	Reference	0.85 (0.66‐1.10)	**0.67 (0.50**‐**0.91)**	**1.04 (1.01**‐**1.08)**
Model 2^b^	Reference	0.93 (0.72‐1.21)	0.87 (0.62‐1.23)	1.01 (0.97‐1.05)
Model 3^c^	Reference	0.89 (0.68‐1.16)	0.82 (0.57‐1.18)	1.02 (0.98‐1.07)
MIH (n* *=* *1780) (Yes vs No)	n* *=* *650 (66 vs 709)	n* *=* *498 (38 vs 498)	n* *=* *507 (42 vs 465)	n* *=* *1780 (146 vs 1634)
OR (95% CI)				
Model 1^a^	Reference	0.87 (0.57‐1.32)	0.92 (0.60‐1.41)	1.04 (0.98‐1.10)
Model 2^b^	Reference	0.92 (0.60‐1.42)	1.15 (0.71‐1.87)	1.02 (0.96‐1.08)
Model 3^c^	Reference	0.85 (0.55‐1.33)	0.99 (0.58‐1.69)	1.05 (0.98‐1.12)

HSPM, hypomineralized second primary molar; MIH, molar incisor hypomineralization.

Values are odds ratios (OR) with 95% confidence interval (CI).

Significant associations are bold.

a Model 1 = adjusted for child's sex, gestational age (mid‐gestational), age of mother, BMI before pregnancy.

b Model 2 = adjusted for all factors in model 1 and additionally adjusted for factors related to enamel hypomineralization (Alcohol use during pregnancy, child's ethnicity, low birth weight and fever in first year of life).

c Model 3 = adjusted for all factors in model 2 and additionally adjusted for factors related to 25(OH)D levels (Household income at intake, educational level mother at intake, folic acid use during pregnancy, parity and season of blood draw).

Children with severely deficient 25(OH)D concentrations in umbilical cord blood serum had significantly lower odds of having HSPMs than children with sufficient to optimal levels (Table 3; OR, 0.63; 95% CI, 0.45‐0.88). Further adjustment for other confounders in models 2 and 3, however, showed no associations. The level of the cord blood serum 25(OH)D concentrations was not associated with the presence of MIH in the 6‐year‐old children.

**Table 3:** Associations of cord blood serum 25(OH)D concentrations with HSPM and MIH

	Cord Blood Serum 25(OH)D concentrations			
	≥50 nmol/L (Sufficient to Optimal)	25‐50 nmol/L (Deficient)	<25 nmol/L (Severely Def.)	Per 10 nmol/L
HSPM (n* *=* *3092) (Yes vs No)	n* = *650 (72 vs 578)	n* = *1106 (109 vs 997)	n* = *1336 (88 vs 1248)	n* *=* *3092 (269 vs 2823)
OR (95% CI)				
Model 1^a^	Reference	0.90 (0.66‐1.23)	**0.63 (0.45**‐**0.88)**	**1.09 (1.03**‐**1.15)**
Model 2^b^	Reference	0.95 (0.69‐1.31)	0.80 (0.56‐1.14)	1.05 (0.99‐1.12)
Model 3^c^	Reference	0.94 (0.67‐1.31)	0.79 (0.53‐1.18)	1.05 (0.98‐1.13)
MIH (n* *=* *1315) (Yes vs No)	n* = *233 (21 vs 212)	n* = *438 (37 vs 401)	n* = *644 (57 vs 587)	n* *=* *1315 (115 vs 1200)
OR (95% CI)				
Model 1^a^	Reference	0.92 (0.53‐1.62)	0.95 (0.55‐1.65)	0.98 (0.89‐1.08)
Model 2^b^	Reference	0.97 (0.55‐1.70)	1.11 (0.62‐1.98)	0.94 (0.84‐1.05)
Model 3^c^	Reference	0.94 (0.52‐1.70)	1.06 (0.55‐2.02)	0.95 (0.84‐1.07)

HSPM, hypomineralized second primary molar; MIH, molar incisor hypomineralization.

Values are odds ratios (OR) with 95% confidence interval (CI).

Significant associations are bold.

a Model 1 = adjusted for child's sex, gestational age (mid‐gestational), age of mother, BMI before pregnancy.

b Model 2 = adjusted for all factors in model 1 and additionally adjusted for factors related to enamel hypomineralization (Alcohol use during pregnancy, child's ethnicity, low birth weight and fever in first year of life).

c Model 3 = adjusted for all factors in model 2 and additionally adjusted for factors related to 25(OH)D levels (Household income at intake, educational level mother at intake, folic acid use during pregnancy, parity and season of blood draw).

Table 4 shows the associations between 25(OH)D concentrations, measured at the age of six, with HSPM and MIH. Model 1 showed that children with a deficient 25(OH)D status had significantly lower odds for having HSPM (OR, 0.73; 95% CI, 0.55‐0.98) than those with optimal levels. After more extensive adjustment in models 2 and 3, this association was no longer apparent. Results for MIH were similar. In model 1, children with a deficient 25(OH)D status had significantly lower odds of having MIH (OR 0.68, 95% CI, 0.53‐0.88) than those with an optimal serum concentration. However, after further adjustment, all ORs were nonsignificant.

**Table 4:** Associations of childhood serum 25(OH)D concentrations with HSPM and MIH

	Serum 25(OH)D concentrations				
	≥75 nmol/L (Optimal)	50‐75 nmol/L (Sufficient)	25‐50 nmol/L (Deficient)	<25 nmol/L (Severely Def.)	Per 10 nmol/L
HSPM (n* *=* *3642) (Yes vs No)	n* = *1254 (124 vs 1130)	n* = *1327 (134 vs 1193)	n* = *843 (59 vs 784)	n* = *218 (12 vs 206)	n* *=* *3642 (329 vs 3313)
OR (95% CI)					
Model 1^a^	Reference	1.05 (0.92‐1.20)	0.73 (0.54‐1.00)	0.59 (0.32‐1.09)	1.03 (0.99‐1.07)
Model 2^b^	Reference	1.12 (0.86‐1.46)	0.88 (0.63‐1.24)	0.84 (0.44‐1.60)	1.00 (0.95‐1.04)
Model 3^c^	Reference	1.20 (0.91‐1.57)	1.02 (0.70‐1.48)	1.03 (0.52‐2.04)	0.97 (0.92‐1.02)
MIH (n* *=* *1556) (Yes vs No)	n* = *459 (45 vs 414)	n* = *548 (42 vs 506)	n* = *431 (27 vs 404)	n* = *118 (9 vs 109)	n* *=* *1556 (123 vs 1433)
OR (95% CI)					
Model 1^a^	Reference	**0.79 (0.63**‐**0.99)**	**0.68 (0.53**‐**0.88)**	0.96 (0.65‐1.43)	1.06 (1.03‐1.10)
Model 2^b^	Reference	0.81 (0.52‐1.26)	0.75 (0.44‐1.27)	1.14 (0.51‐2.57)	1.05 (0.98‐1.13)
Model 3^c^	Reference	0.81 (0.51‐1.29)	0.72 (0.40‐1.31)	1.05 (0.42‐2.61)	1.07 (0.98‐1.16)

HSPM, hypomineralized second primary molar; MIH, molar incisor hypomineralization.

Values are odds ratios (OR) with 95% confidence interval (CI).

Significant associations are bold.

a Model 1 = adjusted for child's sex, age, weight and length.

b Model 2 = additionally adjusted for factors related to enamel hypomineralization (Alcohol use during pregnancy, child's ethnicity, low birth weight and fever in first year of life).

c Model 3 = additionally adjusted for factors related to 25(OH)D levels (Household income, educational level mother, folic acid use during pregnancy, parity, watching television, playing outside, season of blood draw).

In sensitivity analyses, in which we restricted our analyses to a subgroup of children with 25(OH)D data available at all three time points, similar effect estimates were observed as those obtained in the full populations (Table S3). We found no significant interaction between 25(OH)D and child's age, sex or ethnicity for the association with HSPMs or MIH. No better fit of any model was found after applying natural cubic splines (all *P* > .05), indicating linearity to the logit.

## 4 DISCUSSION

Our findings provide no evidence for an association between 25(OH)D status during foetal life, at birth or at age six with the presence of HSPMs or MIH in 6‐year‐olds. Despite a tendency towards lower odds for both HSPMs and MIH in children with lower 25(OH)D concentrations in the basic models, all apparent associations disappeared after adjusting for possible confounders. Furthermore, associations did not differ by child age, sex or ethnicity.

To put the above findings in perspective, some limitations of our study have to be addressed. Children who did not have their first permanent molar yet could not be included, which resulted in smaller sample sizes for MIH in all analyses compared with HSPM. This may have introduced possible selection bias. Children with complete data on MIH were older on average, more often female and more often had a non‐Dutch or other non‐Western background. This may have resulted in underestimation of the MIH‐prevalence, but it is not likely to have biased our results. Ideally, diagnoses of HPSM and MIH were based on clinical examinations, but due to the study setting we had to make use of digital intraoral photographs.^33^ This may have led to an underestimation and/or nondifferential misclassification of pathological findings, resulting in possible information bias. We tried to minimalize the chance of having this bias using a reliable method, but some bias may still be present.^34^ Furthermore, not all children with 25(OH)D measurements had photographs of sufficiently high quality for diagnosing HSPM and/or MIH due to blurriness of the pictures (eg, movements) which have led to excluded children. Sampling bias may therefore have occurred, reducing generalizability to the population, but may not have biased our effect estimates. It is expected that the association between 25(OH)D status and dental enamel hypomineralization is the same for included and excluded participants. Another limitation of our study is that we did not consider different distribution patterns of HSPMs and MIH. This limited the possibility to associate vitamin D status with numerical data. Still, the number of children with HSPMs and MIH would have been the same, as the diagnosis was based on the index teeth as stated in the EAPD‐criteria.^24^ Furthermore, to prevent possible attrition bias, we implemented a multiple imputation method for missing data of covariates. Sensitivity analyses of the imputed data, however, did not result in significant differences in outcome compared to analyses of the original data. The major strength of our study was that we were able to include not only 25(OH)D status when the teeth were already developed, but we have also measured 25(OH)D concentrations at two time points in time during tooth mineralization, which is unique.^22^, ^35^ Another strength of our study was the large and diverse study population of children that could be included and analysed. Furthermore, we were able to adjust for many important factors related to 25(OH)D concentrations, which were not always considered in previous studies.

We were the first to study the association between serum 25(OH)D status and HSPM prevalence in children. Enamel of HSPMs is thought to be less resistant to dental caries and recent studies concluded high 25(OH)D concentrations in children, high prenatal maternal 25(OH)D concentrations, and even higher vitamin D intake during pregnancy, to be associated with lower risk of dental caries in primary dentition.^2^, ^36^, ^37^, ^38^ Given these results, we hypothesized that 25(OH)D deficiency during tooth development would result in weaker enamel or even hypomineralized enamel. Moreover, the odds of having HSPMs is higher in children with a lower bone mass,^21^ which is influenced by vitamin D.^39^ It was therefore unexpected to observe no association between 25(OH)D and HSPM prevalence in 6‐year‐old children. In line with our findings for enamel hypomineralization, however, we also recently observed no association of foetal vitamin D status with children's bone mass.^28^

Kühnisch et al^15^ were the first to have studied the association between a child's serum 25(OH)D status and MIH. Contradictory to their results, we did not find elevated serum 25(OH)D concentrations in children to be negatively correlated with MIH. Compared to their study, we included children that were on average 4 years younger, but it is unlikely that this could explain the discrepancy in results. Despite the inclusion of younger children, we had the same number of MIH‐cases as Kühnisch et al^15^ Furthermore, they reported the lack of earlier 25(OH)D concentration measurements during the period of tooth development as a limiting factor of their study,^15^ because the development of teeth already starts in utero.^22^ We were also able to examine 25(OH)D in a prenatal and early postnatal period. This was a major strength of our study. However, neither the prenatal nor the postnatal 25(OH)D status showed a significant association with HSPMs or MIH.

In conclusion, in this large population‐based cohort, 25(OH)D concentrations in prenatal, early postnatal and later postnatal life are not associated with HPSMs or with MIH at the age of six. To our knowledge, we are the only research group, together with Kühnisch et al^15^ to have studied the association between 25(OH)D status and dental enamel hypomineralization with contradictory results. Therefore, we encourage other cohorts to replicate our findings. Replication in observational studies is needed to confirm whether or not vitamin D supplementation, as a preventive agent against enamel hypomineralization, is worth to be investigated in clinical trials. This could be performed by setting up a cohort or by embedding a study within an existing cohort with repeated and early measurements of serum 25(OH)D in children. Ideally, during the developmental period of teeth. Furthermore, it is important to keep on searching for different preventive possibilities and aetiological factors for dental hypomineralization in children, which still are unknown.^14^ Moreover, despite null findings with hypomineralization, it would be interesting to study the association between 25(OH)D status and dental caries in our population. The pathway in which vitamin D affects the risk of developing dental caries may involve pathways other than enamel mineralization.^40^

## CONFLICT OF INTEREST

The funders had no role in study design, data collection and analysis, decision to publish or preparation of the manuscript. The authors declare no potential conflicts of interest with respect to the authorship and/or publication of this article.
