# Supplementary material for: Foetal, neonatal and child vitamin D status and enamel hypomineralization
Source: Community Dent Oral Epidemiol. 2018 Mar 1;46(4):343–51. doi: 10.1111/cdoe.12372 (PMC6446811; doi:10.1111/cdoe.12372)
Supplement: Supplementary file 1 [file CDOE-46-343-s001.docx]

| **Table S1.** Comparison between participants with complete dental data on MIH and participants with dental photographs, but without information on MIH (n = 4,750)^1^ | | | | | |
| --- | --- | --- | --- | --- | --- |
|  |  | **Complete data (%)** | **No complete data (%)** | | |
| Age child | (n = 4,750) | n = 1,780 (37.5) | n = 2,970 (62.5) | | |
|  | Mean (95% CI) | 6.41 (6.38 to 6.44) | 6.00 (5.99 to 6.01) | | |
| Sex child | (n = 4,750) | n = 1,780 (37.5) | n = 2,970 (62.5) | | |
|  | Boy | 796 (44.7) | 1,565 (52.7) | | |
|  | Girl | 984 (55.3) | 1,405 (47.3) | | |
| Child's ethnicity | (n = 4,651) | n = 1,738 (37.3) | n = 2,913 (62.7) | | |
|  | Dutch & other Western | 1,039 (59.8) | 1,991 (68.3) | | |
|  | Moroccan & Turkish | 313 (18.0) | 339 (11.6) | | |
|  | African | 279 (16.1) | 403 (13.8) | | |
|  | Asian | 107 (6.16) | 180 (6.18) | | |
| Alcohol use during pregnancy | (n = 4,168) | n = 1,542 (37.0) | n = 2,626 (63.0) | | |
|  | never | 767 (49.7) | 1,115 (42.5) | | |
|  | until pregnancy known | 184 (11.9) | 412 (15.7) | | |
|  | continued | 591 (38.3) | 1,099 (41.9) | | |
| Low birth weight | (n = 4,725) | n = 1,773 (37.5) | n = 2,952 (62.5) | | |
|  | No | 1,700 (95.9) | 2,789 (94,5) | | |
|  | Yes | 73 (4.12) | 163 (5.52) | | |
| Fever first year of life | (n = 3,134) | n = 999 (31.8) | n = 2,135 (68.2) | | |
|  | No | 186 (18.6) | 378 (17.7) | | |
|  | Yes | 813 (81.4) | 1,757 (82.3) | | |
| Maternal educational level | (n = 4,462) | n = 1,648 (36.9) | n = 2,814 (63.1) | | |
|  | High | 351 (21.3) | 763 (27.1) | | |
|  | Mid-high | 316 (19.2) | 644 (22.9) | | |
|  | Mid-low | 531 (32.2) | 882 (31.3) | | |
|  | Low | 450 (27.3) | 525 (18.7) | | |
| Household Income | (n = 1,648) | n = 679 (41.2) | n = 969 (58.8) | | |
|  | >€3300 | 300 (44.2) | 483 (49.8) | | |
|  | €2000-€3300 | 252 (37.1) | 339 (35.0) | | |
|  | <€2000 | 127 (18.7) | 147 (15.2) | | |
| Folic acid use | (n = 3,616) | n = 1,317 (36.4) | n = 2,299 (63.6) | | |
|  | Never | 401 (30.4) | 472 (20.5) | | |
|  | Start first ten weeks | 408 (31.0) | 758 (33.0) | | |
|  | Start periconceptional | 508 (38.6) | 1,068 (46.5) | | |
| Parity | (n = 4,702) | n = 1,764 (37.5) | n = 2,938 (62.5) | | |
|  | Mean (95% CI) | 0.61 (0.57 to 0.65) | 0.57 (0.54 to 0.60) | | |
| ^1^Percentages of categorical variables are based on the number of valid cases.  ^2^p-values are based on the Pearson Chi-Square test.  Significant associations are bold. | | | |  |  |
