# Supplementary material for: Foetal, neonatal and child vitamin D status and enamel hypomineralization
Source: Community Dent Oral Epidemiol. 2018 Mar 1;46(4):343–51. doi: 10.1111/cdoe.12372 (PMC6446811; doi:10.1111/cdoe.12372)
Supplement: Supplementary file 3 [file CDOE-46-343-s003.docx]

| **Table S3.** Associations of 25(OH)D concentrations with HSPM and MIH among children with 25(OH)D data available at all three time points | | | | | |
| --- | --- | --- | --- | --- | --- |
|  | | **Mid-gestational Serum 25(OH)D concentration** | | |  |
|  |  | **≥ 50 nmol/L**  **(Sufficient**  **to Optimal)** | **25-50 nmol/L**  **(Deficient)** | **< 25 nmol/L**  **(Severely Def.)** | **Per 10 nmol/L** |
| **HSPM (*n* = 1,698)**  **(Yes vs No)** | | *n =* 879  (89 vs. 790) | *n =* 461  (38 vs. 423) | *n =* 358  (21 vs. 337) | *n* = 1,698  (148 vs. 1,550) |
|  | Model 1*^1^* | Reference | 0.83  (0.56 to 1.25) | 0.63  (0.37 to 1.05) | 1.03  (0.98 to 1.09) |
| OR (95% CI) | Model 2*^2^* | Reference | 0.92  (0.62 to 1.39) | 0.98  (0.55 to 1.73) | 0.99  (0.93 to 1.05) |
|  | Model 3*^3^* | Reference | 0.88  (0.57 to 1.34) | 0.89  (0.48 to 1.67) | 1.00  (0.93 to 1.07) |
| **MIH (*n* = 702)**  **(Yes vs No)** | | *n =* 309  (24 vs. 285) | *n =* 221  (16 vs. 205) | *n =* 172  (14 vs. 158) | *n* = 702  (54 vs. 648) |
|  | Model 1*^1^* | Reference | 0.91  (0.47 to 1.78) | 1.01  (0.49 to 2.10) | 1.05  (0.96 to 1.16) |
| OR (95% CI) | Model 2*^2^* | Reference | 0.95  (0.48 to 1.87) | 1.29  (0.56 to 2.97) | 1.04  (0.93 to 1.16) |
|  | Model 3*^3^* | Reference | 0.89  (0.43 to 1.82) | 1.34  (0.52 to 3.46) | 1.05  (0.93 to 1.18) |
|  | | **Cord Blood Serum 25(OH)D concentration** | | |  |
|  | | **≥ 50 nmol/L**  **(Sufficient**  **to Optimal)** | **25-50 nmol/L**  **(Deficient)** | **< 25 nmol/L**  **(Severely Def.)** | **Per 10 nmol/L increase** |
| **HSPM (*n* = 1,698)**  **(Yes vs No)** | | *n =* 365  (40 vs. 325) | *n =* 616  (63 vs. 553) | *n =* 717  (45 vs. 672) | *n* = 1,698  (148 vs. 1.550) |
|  | Model 1*^1^* | Reference | 0.94  (0.62 to 1.43) | **0.60**  **(0.38 to 0.96)** | 1.06  (0.98 to 1.14) |
| OR (95% CI) | Model 2*^2^* | Reference | 0.99  (0.65 to 1.52) | 0.80  (0.49 to 1.29) | 1.00  (0.92 to 1.09) |
|  | Model 3*^3^* | Reference | 0.97  (0.62 to 1.53) | 0.76  (0.44 to 1.33) | 1.00  (0.90 to 1.10) |
| **MIH (*n* = 702)**  **(Yes vs No)** | | *n =* 117  (10 vs. 107 | *n =* 237  (18 vs. 219) | *n =* 348  (26 vs. 322) | *n* = 702  (54 vs. 648) |
|  | Model 1*^1^* | Reference | 0.84  (0.37 to 1.90) | 0.80  (0.36 o 1.79) | 0.98  (0.84 to 1.15) |
| OR (95% CI) | Model 2*^2^* | Reference | 0.90  (0.39 to 2.04) | 0.96  (0.41 to 2.22) | 0.94  (0.79 to 1.11) |
|  | Model 3*^3^* | Reference | 0.81  (0.34 to 1.95) | 0.77  (0.29 to 2.03) | 0.94  (0.77 to 1.15) |
| Values are odds ratios with 95% confidence interval (CI)  ^1^ Model 1 = adjusted for child’s sex, gestational age of birth, age of mother, BMI before pregnancy;  *^2^* Model 2 = adjusted for all factors in model 1 and additionaly adjusted for factors related to enamel hypomineralization (Alcohol use during pregnancy, child’s ethnicity, low birth weight and fever in first year of life);  ^3^ Model 3 = adjusted for all factors in model 2 and additionally adjusted for factors related to 25(OH)D levels (Household income at intake, educational level mother at intake, folic acid use during pregnancy, parity, season of blood draw, and serum 25(OH)D levels at 6 years of age);  Signifcant associations are **bold**.  HSPM = Hypomineralized Second Primary Molar; MIH = Molar Incisor Hypomineralization. | | | | | |
